# Peer Review of "Are We Sure We Fully Understand What an Infodemic Is? A Global Perspective on Infodemiological Problems"

**DOI:** 10.2196/40303

**Published:** 2022-07-21

**Keywords:** communication, conspiracy, COVID-19, education, fake news, infodemic, infodemiology, mass media, public health, risk perception, science

*This is a peer-review report submitted for the paper “Are We Sure We Fully Understand What an Infodemic Is? A Global Perspective on Infodemiological Problems.”*

## Round 1 Review

### General Comments

This paper [1] aims to highlight principal problems related to the understanding of the infodemic phenomenon. Although it debates interesting aspects of this field, the paper seems incomplete and evasive. Moreover, the authors used problematic definitions for distinct types of information disorder.

### Specific Comments

#### Major Comments

1. The neologism “dismisinformation” is problematic; we commonly use “misinformation” as an umbrella term when we cannot distinguish the type of information disorder.
2. The definition of fake news is advancing toward a specific information disorder, that is, it is not a mere simplification of phenomena (see, for instance, Molina et al [2]).
3. The authors affirm that infodemics cannot exist without dismisinformation. This sentence is imprecise because information disorder also includes malinformation, fake news, and conspiracy theory. The background adopted by the authors to reflect on the presented problems can be compromised by such misconceptions.
4. I recommend that the authors concentrate their efforts on a specific problem, presenting a deep argumentation about the mechanisms that contribute to the success of information disorder during the pandemic.
